# Acceptance of Social Media Recruitment for Clinical Studies Among Patients With Hepatitis B: Mixed Methods Study

**DOI:** 10.2196/54034

**Published:** 2024-08-26

**Authors:** Theresa Willem, Bettina M Zimmermann, Nina Matthes, Michael Rost, Alena Buyx

**Affiliations:** 1 Institute of Molecular Immunology Klinikum Rechts der Isar, TUM School of Medicine and Health Technical University of Munich Munich Germany; 2 Institute of History and Ethics in Medicine TUM School of Medicine and Health Technical University of Munich Munich Germany; 3 Department of Science, Technology and Society (STS) School of Social Sciences and Technology Technical University of Munich Munich Germany; 4 Institute of Philosophy Multidisciplinary Center for Infectious Diseases University of Bern Bern Switzerland; 5 Institute for Biomedical Ethics University of Basel Basel Switzerland

**Keywords:** Facebook, Twitter, social media, clinical trial, enrollment, health technology acceptance, ethics, infectious diseases, privacy, data protection, stigma, discrimination

## Abstract

**Background:**

Social media platforms are increasingly used to recruit patients for clinical studies. Yet, patients’ attitudes regarding social media recruitment are underexplored.

**Objective:**

This mixed methods study aims to assess predictors of the acceptance of social media recruitment among patients with hepatitis B, a patient population that is considered particularly vulnerable in this context.

**Methods:**

Using a mixed methods approach, the hypotheses for our survey were developed based on a qualitative interview study with 6 patients with hepatitis B and 30 multidisciplinary experts. Thematic analysis was applied to qualitative interview analysis. For the cross-sectional survey, we additionally recruited 195 patients with hepatitis B from 3 clinical centers in Germany. Adult patients capable of judgment with a hepatitis B diagnosis who understood German and visited 1 of the 3 study centers during the data collection period were eligible to participate. Data analysis was conducted using SPSS (version 28; IBM Corp), including descriptive statistics and regression analysis.

**Results:**

On the basis of the qualitative interview analysis, we hypothesized that 6 factors were associated with acceptance of social media recruitment: using social media in the context of hepatitis B (hypothesis 1), digital literacy (hypothesis 2), interest in clinical studies (hypothesis 3), trust in nonmedical (hypothesis 4a) and medical (hypothesis 4b) information sources, perceiving the hepatitis B diagnosis as a secret (hypothesis 5a), attitudes toward data privacy in the social media context (hypothesis 5b), and perceived stigma (hypothesis 6). Regression analysis revealed that the higher the social media use for hepatitis B (hypothesis 1), the higher the interest in clinical studies (hypothesis 3), the more trust in nonmedical information sources (hypothesis 4a), and the less secrecy around a hepatitis B diagnosis (hypothesis 5a), the higher the acceptance of social media as a recruitment tool for clinical hepatitis B studies.

**Conclusions:**

This mixed methods study provides the first quantitative insights into social media acceptance for clinical study recruitment among patients with hepatitis B. The study was limited to patients with hepatitis B in Germany but sets out to be a reference point for future studies assessing the attitudes toward and acceptance of social media recruitment for clinical studies. Such empirical inquiries can facilitate the work of researchers designing clinical studies as well as ethics review boards in balancing the risks and benefits of social media recruitment in a context-specific manner.

## Introduction

### Benefits and Risks of Using Social Media Recruitment for Clinical Studies

Recruiting clinical study participants through social media has the potential to increase the recruitment accrual in a cost-effective way [1]. Consequently, social media recruitment has been increasingly applied for clinical studies, often in parallel with other recruitment strategies. However, social media recruitment still bears a host of challenges. First, maintaining a social media presence and community management can be resource intensive. Second, when used as a stand-alone recruiting method, it might yield a cohort of limited demographic representativeness. Finally, social media recruitment comes with ethical issues, particularly when used to recruit for clinical studies [2]. Because social media recruitment includes reaching potential research participants outside a clinical setting and in a public online space without direct personal contact, risks related to social stigma, privacy infringement, loss of trust, and psychological harm have been discussed [3]. To mitigate some of these risks, prioritizing investigator transparency and obtaining explicit consent when recruiting from others’ social network was suggested [4]. Yet, because the activities of social media platforms are primarily unregulated and partly belong to large global technology companies, activities conducted on social media, including study recruitment, can never be fully controlled by researchers or institutions. Remaining privacy-infringing risks include hidden data collection and profiling, particularly problematic for patients carrying vulnerable characteristics [5].

Early studies assessing social media recruitment for clinical studies focused on the effectiveness of the method. For example, Frandsen et al [3] used social media recruitment for a smoking cessation trial and compared their cohort recruited from a Facebook-based approach to cohorts resulting from other recruitment methods. They found no differences between the cohorts regarding socioeconomic or smoking characteristics, except that participants recruited via Facebook were significantly younger. Wisk et al [4] recruited college students with type 1 diabetes, a hard-to-reach population, using a variety of outreach channels, including social media. They found that Facebook was the most successful recruitment method. Guthrie et al [5] found that Facebook advertising was significantly cheaper than recruiting via mail. While these studies allow insights into the utility of social media recruitment from the perspective of researchers, studies assessing patients’ perspectives and attitudes toward social media for clinical study recruitment are lacking. This study aims to deliver first evidence on patient attitudes toward social media recruitment, focusing on patients with hepatitis B.

### Patients With Hepatitis B and Social Media

Patients with hepatitis B are a particularly interesting cohort to study acceptance for social media recruitment as the particularities of the disease exhibit potentially confounding factors for their attitudes toward social media recruitment. First, there is robust empirical evidence that patients with hepatitis B can be subject to social stigma [6-10]. Therefore, the risk of public exposure to hepatitis B diagnosis on social media renders them—and patients with other stigmatized traits and conditions—particularly vulnerable in the context of social media recruitment [11]. Second, hepatitis B in Europe is particularly prevalent in certain immigrant populations, which are at risk of being neglected for clinical studies due to language barriers and lack of health care access. Social media recruitment can help include patient populations who otherwise would be disregarded for clinical studies or are hard to reach [12-14].

### Study Rationale and Objectives

However, the effectiveness of social media recruitment crucially hinges on technology acceptance. To date, the attitudes of patients regarding social media recruitment are underexplored. Addressing this gap, this mixed methods study assesses factors predicting the acceptance of social media recruitment among patients with hepatitis B. On the basis of qualitative individual interviews with 6 patients with hepatitis B and 30 multidisciplinary experts and a literature review, we hypothesized that general social media use (hypothesis 1), social media literacy (hypothesis 2), interest in clinical studies (hypothesis 3), trust (hypothesis 4), privacy needs (hypothesis 5), and perceived stigma (hypothesis 6) are associated with acceptance of social media recruitment.

## Methods

### Study Design

This study is part of the European Union–funded international research consortium “TherVacB—A Therapeutic Vaccine to Cure Hepatitis B,” work package 6 (ethical, legal, and social aspects of social media recruitment). Using a mixed methods design, we first conducted an explorative qualitative multistakeholder interview study assessing the ethical, legal, social, and practical implications of social media recruitment for clinical studies [2]. The hypotheses investigated in this paper are based on these interviews and a conceptual literature review mapping the ethical implications of social media recruitment [11]. The reporting of this study followed the Strengthening the Reporting of Observational Studies in Epidemiology guidelines [15].

### Survey Recruitment

On the basis of preliminary statistical power analysis and pragmatic considerations of available study participants, we aimed for 200 responses in a recruitment period of 7 months. Due to administrative constraints, including the COVID-19 pandemic, the overall recruitment period was prolonged by 5 months (total recruitment period 12 months, June 4, 2022, to May 31, 2023), and the recruitment period varied among the recruiting clinics (Multimedia Appendix 1).

Adult, German-speaking patients diagnosed with acute or chronic viral hepatitis B were recruited from 3 large university hospitals in Germany. We chose such a venue-based recruitment methodology because it is considered one of the best options to recruit representative samples from hard-to-reach populations [16]. The clinical staff was instructed to distribute the study information leaflet to every eligible patient in the study period, explaining the implications of the study and inviting them to fill out the questionnaire. To limit recruitment bias and enhance sample representativeness, study nurses were briefed to avoid self-selected restrictions in recruitment and, if possible, to give a questionnaire to every incoming patient with hepatitis B who understood German sufficiently well. However, because of the administrative burden of the clinical staff, only 30.4% (285/939) of the estimated eligible incoming patients received the questionnaire (Multimedia Appendix 1). Because this low distribution number results from administrative burden in the clinic, we do not expect this to have a relevant impact on representativeness (refer to the Limitations subsection under the Discussion section). Completed questionnaires (207/285, 72.6% of the distributed questionnaires; Multimedia Appendix 1) were collected in the recruiting hospital and sent to the authors via mail.

### Survey Construction

The dependent variable (acceptance of social media recruitment) was constructed based on the Technology Acceptance Model [17,18], involving the dimensions of perceived usefulness; perceived ease of use, intentions, and problem awareness; and proved good internal consistency (Cronbach α=0.863). Possible predictors for social media recruitment acceptance were identified based on the abovementioned hypotheses and operationalized by, if possible, existing validated questionnaires. For 3 (33%) of the 9 independent variables, we used existing validated questionnaires that were found to be of excellent reliability: the social media literacy scale (14 items, Cronbach α=0.947) [19], the Berger HIV Stigma Scale for use among patients with hepatitis C virus (6 items, Cronbach α=0.931) [20], and the Privacy Attitude Questionnaire [21]. For the latter, we included a shortened version that covered the dimensions developed in the Privacy Attitude Questionnaire but targeted it toward the hepatitis B context. From these dimensions, 2 subscales were created: secrecy of hepatitis B diagnosis (2 items, Cronbach α=0.623) and data privacy needs regarding hepatitis B diagnosis (2 items, Cronbach α=0.587).

For the remaining variables, no validated tools existed. Hence, we developed new scales for each variable of interest. As indicated by internal consistency, these were of moderate, good, or excellent reliability: general social media use (8 items, Cronbach α=0.676), hepatitis B–related social media use (6 items, Cronbach α=0.906), interest in clinical studies (2 items, Cronbach α=0.895), and trust in information sources regarding hepatitis B (11 items, Cronbach α=0.905; 2 subscales were created: trust in medical information sources—4 items, Cronbach α=0.784 and trust in nonmedical information sources, ie, traditional media, social media, other patients, poster advertisements, etc—7 items, Cronbach α=0.881). In addition to these adapted and self-developed scales, we included 4 demographic variables in the regression model (age, gender, education, and mother tongue as an indicator of migration background). A preliminary version of the questionnaire was discussed with 3 experts from the fields of infectiology and bioethics and then adapted and shortened based on their comments. We then performed cognitive pretesting [22] with 6 patients with hepatitis B, leading to minor changes. The full questionnaire is provided in Multimedia Appendix 2.

### Statistical Analysis

Using SPSS (version 28.0; IBM Corp), we (1) performed descriptive analyses, (2) determined independent factors associated with participants’ acceptance of social media as a recruitment tool for clinical hepatitis B studies through multiple linear regression analysis, and (3) performed additional exploratory bivariate analyses of hepatitis B–related stigma (ie, correlation, independent 2-tailed *t* test). The statistical significance level was set at *P*<.05. For multiple linear regression analysis, assumption checks were performed before the interpretation of the model (Multimedia Appendix 3). For the scale measuring the frequency of social media use, missing values were replaced by “0” (ie, “never”), assuming that participants did not tick a box, as they did not know the respective social media platform. Overall, 71.3% (139/195) of the participants completed all items, resulting in 3.66% (478/13,065) missing values and 81% (54/67) incomplete variables.

For the linear regression analysis, theoretical considerations and hypotheses derived from our previous qualitative study determined predictor selection. In addition, the sample size or predictor ratio a priori determines variable selection for regression modeling. According to Harrell [23], a fitted regression model is likely to be reliable when p<m/10 or p<m/20 (average requirement: p<m/15), where p is the number of predictors and m is the sample size. Applying this requirement to our sample size (N=195) and having missing data, we preliminarily limited the number of included predictors to 11. The following 11 predictors were included in the regression model: general social media use, social media literacy, hepatitis B–related social media use, interest in clinical studies, trust in medical information sources regarding hepatitis B (dichotomized to meet assumption of linearity), trust in nonmedical information sources regarding hepatitis B, secrecy of hepatitis B (dichotomized to meet assumption of linearity), data privacy needs regarding hepatitis B (dichotomized to meet assumption of linearity), perceived stigma, age, and education. Assumptions checks for regression analyses are presented in Multimedia Appendix 3.

### Ethical Considerations

For study consent, participants were asked to confirm having read and understood the study information and to consent to the study participation by checking a consent box at the beginning of the questionnaire. Only questionnaires with this box checked were included in the analysis (12/207, 5.8% of the questionnaires were excluded for that reason; Multimedia Appendix 1). The ethics committees from the Technical University of Munich (12/22-S-NP), Hannover Medical School (10368_BO_K_2022), and University Clinic Leipzig (189/22-lk) approved the study.

## Results

### Deriving Hypotheses

#### Overview

After conducting an in-depth literature review on the ethical and social challenges surrounding social media recruitment for clinical studies [11], we developed 2 semistructured interview guides, one targeted at patients with hepatitis B and the other targeted at multidisciplinary experts. On the basis of interviews with 6 patients that were triangulated with findings from 30 interviews with experts, we qualitatively assessed what factors could be associated with the acceptance of social media recruitment for clinical hepatitis B studies. On the basis of these findings, we derived hypotheses to be tested quantitatively in a survey among patients with hepatitis B in Germany (Textbox 1).

**Hypotheses derived from qualitative interviews regarding factors potentially associated with the acceptance of social media recruitment for clinical studies among patients with hepatitis B.**

**Hypotheses**

- Hypothesis 1: The more patients use social media for hepatitis B, the higher their acceptance of using social media as a recruitment tool for clinical hepatitis B studies.
- Hypothesis 2: Digital literacy is associated with social media acceptance.
- Hypothesis 3: The higher the general interest in clinical study participation, the higher the acceptance of social media recruitment for clinical studies.
- Hypothesis 4: The more patients trust information sources for hepatitis B, the higher their acceptance of social media recruitment.
- Hypothesis 5: The more patients value privacy, the lower their acceptance of using social media as a recruitment tool for clinical hepatitis B studies.
- Hypothesis 6: The higher the perceived stigma of patients with hepatitis B, the lower their acceptance of social media as a recruitment tool for clinical studies.

#### Intensity of Using Social Media in the Context of Hepatitis B

Most of the patients we talked with were rejecting the idea of being recruited for a clinical hepatitis B study via social media. However, patients who were more actively involved in their own recruitment tended to have more accepting attitudes. For example, patients who described using social media as a tool for informing themselves about potential clinical studies related to their disease were less opposed to being recruited via the same channel. One patient included search engines in their definition of social media and mentioned the following:

You can also advertise on Google. That is quasi/I think it’s better if I [as a patient] search for a study. For example, I search for a study related to psoriasis and enter that term in Google—when the advertisement for a psoriasis study is then made so that it shows up as the first suggestion...I think that’s better because in these instances I’m already searching, so I take the first step, I search for the study. And then the study, or the advertisement must be done in such a way that I can find it. So, I take the first step and then I land on the study.

Patient 3

Similarly, patients who joined shared interest groups, such as patient groups on Facebook, which gather people who deliberately want to share their own experiences with the disease and learn from others’ experiences, were more open toward the idea of being approached and recruited within such groups.

These insights indicate that patients who were already active on social media and found it useful for their personal disease management were more open to being recruited via social media. This led us to the following hypothesis: (H1) The more patients use social media (for hepatitis B), the higher their acceptance of using social media as a recruitment tool for clinical (hepatitis B) studies.

#### Digital Literacy

The patients we interviewed represented a variety of levels regarding social media literacy. While some patients have had very limited contact with social media, others were very active on social media. One patient even described social media content management as part of their daily job. Another had conducted a research web-based questionnaire for which they were recruiting on the web. Analyzing the interviewees’ accounts about their experience with social media, and partially their use habits, we found a scattered connection to social media recruitment acceptance: those who were considered to have higher digital literacy skills were, in some instances, likely to accept social media as a recruitment tool for clinical hepatitis B studies because they perceived other forms of recruitment as outdated:

I think we are living in a time that you have to use social media because if you don’t use it...sending a letter or put[ting it] in the newspaper, will not help you.

Patient 6

On the other end of the spectrum, however, patients with very low digital literacy skills and relatedly very little reported use of social media, or digital media in general, in some instances had difficulties delimiting the concept of social media as such. Presumably, their less nuanced understanding of social media as a concept makes them less strictly opposed to being recruited for a clinical study via social media. One patient, for example, favored personal contact for study recruitment at first but then revised their statement and reported that being helped was even more important than personal contact:

Yes definitely. If it was something important it would be best if we met at a clinic, or I don’t know where this study is being done.... But even via Facebook or Messenger.... Yeah, actually never mind, I don’t care actually.

Patient 2

While the interviews suggested a connection between the acceptance of social media recruitment for clinical hepatitis B studies and digital literacy, it remained unclear whether acceptance was higher with high or low digital literacy. Consequently, we formulated the nondirectional hypothesis that (H2) digital literacy is associated with social media acceptance (SMA).

#### Interest in Clinical Studies

Some participating patients expressed particularly high interest in participating in clinical studies about hepatitis B. One patient explained to us that they were “very, very happy to support studies” (patient 5), and another patient told us the following: “I actually want to help. So, that’s why I get in” (patient 6). Patients like this, who reported an increased willingness to participate in clinical studies in general, seemed more susceptible to social media as a recruitment tool, too.

Another patient perceived it as beneficial that online recruitment made them less dependent on their physician to refer them to the study:

I don’t know if my physician is even internet-savvy, he’s a bit older. And well, then I thought, I have to see for myself because I’m not sure how competent he is with such things. What I mean is, it would be nicer if I...could google for [a clinical trial], land on a platform, search for [relevant studies], see all the information and can get in touch right away and say: “Hey, I am interested in your study. I would like to participate.” Because in my case, the...specialists didn’t even know that this [study] existed.... That’s stupid and got me pretty upset.”

Patient 3

None of the patients interviewed reported that they were generally against participation in clinical studies. This is likely a recruitment bias of this qualitative interview study, which made it difficult to interrogate if patients who are less accepting of clinical studies are also less accepting of social media recruitment. Yet, based on the apparent influence of this aspect in 2 (33%) of 6 patient interviews, we formulated the following hypothesis: (H3) The more patients are interested in clinical studies, the more they accept social media as a recruitment tool for clinical hepatitis B studies.

#### Trust

The role of trust in health care professionals, social media platforms, and other recruitment channels was a very salient aspect of all interviews. Illustrating this, one participating patient with hepatitis B stated the following as a reason for being against social media recruitment:

I just feel such a distrust of social media. Any information I share there, I’m not completely comfortable with/It’s just not a safe way for me to share information.

Patient 4

Other patients were more open to social media recruitment if they knew the source of the advertisement and assigned relevant expertise to them:

It would be okay for me [if someone would contact me on social media to ask whether I would like to meet for a clinical study, as long as] the person is qualified in that direction and is well versed in this expertise.

Patient 2

[R]ecruiting is normally working if the person that suggests it is a person that you trust or you know. So because she was a person I knew from [redacted], then I clicked the link and I got in. Normally we know, of course, that social media is also a trap for many, I don’t know, viruses and this kind of thing. So you don’t open everything if you don’t trust the link.... If I would see it on, I don’t know, social media and as we know, because you have these cookies that you accept, then immediately, they know that you have something or you are looking for some article. Then this kind of things will pop up. Again, it’s all about trusting links. I’m not sure how much I will get in something that is suggesting from just because I click on a link.

Patient 6

More implicitly, another patient emphasized that the clinical setting was the place for them to discuss things in the context of hepatitis B, not social media:

This channel through the [clinic in Germany]... I have a very good opinion of the hospital and I have always been well taken care of there. That is the only channel through which I would talk about my condition and about my/yes.

Patient 1

We analyze the aspect of trust in a separate publication (Willem, T, et al, unpublished data, January 2024) in detail and hypothesize the following: (H4) The more patients trust information sources, the higher their acceptance of social media recruitment. The hypothesis was operationalized for trust in medical information sources (H4a) and trust in nonmedical information sources (H4b).

#### Privacy

A particular concern of most patients we spoke with was their privacy. Privacy is a multifaceted and complex concept, and we found that participants referred to different dimensions of privacy: (1) *data privacy,* defined as the general attitude toward protective measures that empower patients or users to make their own decisions about who can process their data for which purpose; and (2) privacy related to the *perceived secrecy* of the hepatitis B diagnosis.

First, regarding data privacy, several patients perceived recruitment via social media as dubious and suspected some form of data leakage or malicious data collection goals behind the reach outs. This view applied irrespectively to how they would be approached on social media (eg, advertisement banners in their social media timelines or personal contact requests via social media messengers by health care professionals). For example, a patient who reported on being in the process of decreasing their social media use to protect their privacy also said that if someone contacted them on social media regarding clinical study participation, they would “find that very strange, because [I] would ask [my]self, where did they get this information?” and reported that they would feel that this “would rob quite a lot of privacy” (patient 5). Another patient, who reported using WhatsApp as their only social media, explained that by saying that they “consider social media to be useful in some instances;” however, they continued, “It’s too risky for me with my private data and so much advertising. This, for me, trumps all advantages of social media recruitment” (patient 4).

Regarding the second privacy dimension, secrecy, several patients commented on their hepatitis B diagnosis being a very private, intimate matter:

This condition is in my most private, intimate sphere…. And you might be right, I never thought about it in this way, but [my avoiding engaging on social media regarding hepatitis B] may be related to the fact that content I pass on via WhatsApp can be passed on thousands of times with one click.

Patient 1

One patient replied to a question regarding their attitude toward being contacted by a study center via social media that they “would find that difficult”. As a reason, this patient explained the following:

[T]hat’s just the problem: it ends up on social media. See, if someone writes: “Hey, I would like to ask you about your hepatitis B, whether you would participate in a study?” Then this information is out there on social media.... That’s why I had a very, very good feeling when my doctor approached me about [this interview study] and that it just went through the clinic. If she had said, “Look, someone is approaching you via social media,” or something, then I would have said no, right? Because I wouldn’t have wanted to, because these data/social media make money because they have data. They run the ads based on your data and what you type in there or what you say or whatever. And I don’t want that associated with my disease.

Patient 5

These findings led us to the following hypothesis: (H5) The more patients value privacy, the lower their acceptance of using social media as a recruitment tool for clinical hepatitis B studies. The hypothesis was operationalized for secrecy (H5a) and data privacy (H5b).

#### Perceived Stigma

Several interviewed patients with hepatitis B reported fear of being stigmatized if their social environment found out about their diagnosis as an important reason against social media recruitment. One patient, who mentioned that only their closest family members knew about their diagnosis, expressed fear that other people learning the diagnosis would lead to social exclusion:

A broken leg or surgery on the knee or hip. This is apparent to everyone. And everyone assumes that it will heal at some point and that there is no potential infectious danger from these people. Whereas in the case of infectious diseases, no one can assess that, and people get socially excluded very quickly.... And this is why I am so cautious with my data.

Patient 1

A similar view was shared by patient 5. Another patient added that perception of stigma differed depending on the context:

I come from [Eastern European country], I have moved to Germany. So here the mentality is a little bit different. If you say to someone, I have Hepatitis, he is okay with it. He says: “Oh, is not a problem. Normally here we are vaccinated against it.” If you are going to [Eastern European country] and say: “I have Hepatitis B,” it’s like you have a huge disease that can just be taken by a handshake [laughs]. And so I think that’s why I’m going on the conservative site.

Patient 6

The connection between the stigma connected to hepatitis B and the social media–connected perceived privacy risks established by several interview participants led us to the following hypothesis: (H6) The higher the perceived stigma of patients, the lower their acceptance of social media as a recruitment tool for clinical hepatitis B studies.

### Survey Results

#### Participant Characteristics

A total number of 195 eligible questionnaires were included in the statistical analysis of the survey study. Table 1 displays the characteristics of the patients with hepatitis B who participated in the study: more than half of the participants (108/195, 55.4%) were aged between 30 and 49 years. Just above half (110/195, 56.4%) reported having lower educational degrees than Abitur (German equivalent to a high school degree). More than half of the participants (111/195, 56.9%) had another mother tongue than German (only). All participants had a chronic hepatitis B infection, as per the inclusion criterion of this study.

**Table 1:** Participant characteristics (N=195).

Characteristics			Participants, n (%)
**Gender**			
	Male	101 (51.8)	
	Female	88 (45.1)	
	No answer	6 (3.1)	
**Age (y)**			
	18-29	16 (8.2)	
	30-39	50 (25.6)	
	40-49	58 (29.7)	
	50-59	38 (19.5)	
	>60	24 (12.3)	
	No answer	9 (4.6)	
**Education: high school diploma**			
	Yes	71 (36.4)	
	No	110 (56.4)	
	No answer	14 (7.2)	
**Mother tongue (multiple answers possible)**			
	German	101 (51.8)	
	Other	111 (56.9)	
	No answer	12 (6.2)	

#### Description of Scales

The questionnaire included 7 scales that were measured through several items (Table 2 and Multimedia Appendices 1 and 4).

The level of acceptance for social media recruitment was measured through the SMA scale, which was calculated based on 4 questionnaire items (P6.01 to P6.04; Multimedia Appendix 4). Each item was measured by a 5-point Likert scale, ranging from 0 (completely disagree) to 4 (completely agree). Items P6.01 (“Social media are well suited to make patients aware of studies on new hepatitis B treatments”) and P6.02 (“Social media increase the likelihood of success in hepatitis B clinical trials”) formed the subscale of the perceived usefulness of social media recruitment and received moderate agreement (P6.01: mean 1.99, SD 1.23; P6.02: mean 1.81, SD 1.12). Items P6.03 and P6.04 formed the SMA subscale on the perceived usefulness of social media recruitment. Item P6.03 (“I would be recruited via social media for a hepatitis B clinical trial”) received particularly low acceptance (mean 1.13, SD 1.13; Multimedia Appendix 4). P6.04 (I would use social media to learn about hepatitis B clinical trials) received a higher mean acceptance score than P6.03 (mean 1.58, SD 1.23; Multimedia Appendix 4).

The overall SMA score was calculated by summarizing the scores from items 6.01 to 6.04 and ranged from 0 (no acceptance) to 16 (full acceptance; mean 6.48, SD 3.03; Table 2). While 28.7% (56/195) of the respondents rejected social media recruitment with an SMA score of <5, only 10.2% (20/195) of the respondents accepted social media recruitment with an SMA score of >11 (Table 3).

**Table 2:** Description of scales.

	Valid, n (%)	Items, n (%)	Scale, median (range^a^)	Values, mean (SD)
General social media use	195 (100)	8 (15)	11 (0-32)	11.22 (6.51)
Social media literacy (hypothesis 2)	174 (89.2)	14 (25)	41 (0-56)	37.58 (14.60)
Hepatitis B–related social media use (hypothesis 1)	181 (92.8)	6 (11)	3 (0-24)	5.22 (5.61)
Interest in clinical studies (hypothesis 3)	187 (95.9)	2 (4)	6 (0-8)	5.53 (2.45)
Trust in medical information sources	180 (92.3)	4 (7)	11 (0-16)	10.27 (3.64)
Trust in nonmedical information sources (hypothesis 4)	175 (89.7)	7 (13)	8.5 (0-28)	8.36 (5,76)
Acceptance of social media recruitment (dependent variable)	178 (91.3)	4 (7)	6 (0-16)	6.48 (3.93)
Secrecy (hypothesis 5a)	185 (94.9)	2 (4)	2 (0-8)	2.25 (2.09)
Data privacy (hypothesis 5b)	186 (95.4)	2 (4)	7 (0-8)	6.25 (2.10)
Perceived stigma (hypothesis 6)	180 (92.3)	6 (11)	3.5 (0-24)	5.52 (6.02)

^a^Items were measured through a 5-point Likert scale, ranging from 0 (completely disagree) to 4 (completely agree).

**Table 3:** Social media acceptance among patients with hepatitis B in Germany. The higher the score, the higher the acceptance (n=195).

Social media acceptance score	Responses, n (%)
0	20 (10.3)
1	4 (2.1)
2	6 (3.1)
3	8 (4.1)
4	18 (9.2)
5	14 (7.2)
6	20 (10.3)
7	20 (10.3)
8	17 (8.7)
9	12 (6.2)
10	8 (4.1)
11	11 (5.6)
12	7 (3.6)
13	7 (3.6)
14	2 (1)
15	1 (0.5)
16	3 (1.5)
Missing	17 (8.7)

#### Regression Analysis

Using multiple linear regression analyses, we evaluated the predictors of participants’ acceptance of social media as a recruitment tool for clinical hepatitis B studies. Testing the statistical significance of the overall model fit, the *F* test indicated that the predictors included in the model substantially contributed to the explanation of the dependent variable (Table 4). Regression analysis revealed that social media use for hepatitis B, interest in clinical studies, trust in nonmedical information sources, and hepatitis B secrecy independently predicted acceptance of social media as a recruitment tool for clinical hepatitis B studies. More precisely, the higher the social media use for hepatitis B, the higher the interest in clinical studies, the more trust in nonmedical information sources, and the less secret hepatitis B, the higher the acceptance of social media as a recruitment tool for clinical hepatitis B studies (Table 4).

**Table 4:** Multiple linear regression analysis^a^.

	Unstandardized coefficients B (SE)	β	*t* test (*df*)	*P* value	Tolerance	VIF^b^
Constant	4.007 (1.935)	—^c^	2.071 (127)	.04	—	—
General social media use	0.060 (0.051)	.098	1.175 (127)	.24	.628	1.593
Social media literacy	–0.002 (0.025)	–.008	–0.096 (127)	.92	.600	1.668
Hepatitis B–related social media use	0.279 (0.053)	.391	5.299 (127)	<.001	.804	1.234
Interest clinical studies	0.283 (0.127)	.171	2.217 (127)	.03	.732	1.366
Trust medical information sources	–0.601 (0.683)	–.079	–0.879 (127)	.38	.546	1.830
Trust in nonmedical information sources	0.252 (0.058)	.359	4.307 (127)	<.001	.632	1.583
Secrecy	–1.299 (0.542)	–.171	–2.399 (127)	.02	.861	1.161
Data privacy	–0.765 (0.577)	–.099	–1.326 (127)	.19	.792	1.262
Perceived stigma	–0.003 (0.048)	–.004	–0.057 (127)	.95	.770	1.299
Age	–0.052 (0.028)	–.151	–1.842 (127)	.07	.648	1.543
Education	0.770 (0.567)	.102	1.357 (127)	.18	.782	1.278

^a^Overall model fit: *F*_11,127_=9.221, *P*<.001; *R^2^*=0.444; N=139.

^b^VIF: variance inflation factor.

^c^Not applicable.

## Discussion

### Principal Findings

We present the first empirical study investigating how adult patients with hepatitis B accept social media recruitment for clinical studies. Social media have been suggested to increase recruitment accrual, particularly for hard-to-reach populations [13,14,24]. Our study provides a more fine-grained contextualization of this potential. We find that acceptance of social media recruitment among patients with hepatitis B is associated with higher ongoing activity on social media with regard to hepatitis B (confirming H1), a generally high interest in participating in clinical studies for hepatitis B (confirming H3), and high trust recruitment channels outside the clinical setting (confirming H4a). Patients with these characteristics are, consequently, recruitable via social media under the assumptions that (1) patients are most effectively recruited via social media if they accept this channel as a recruitment method and (2) people who do not accept this recruitment channel should also not be recruited in this way.

Yet, 54 (27.7%) out of 195 participants reported an acceptance score of <5 and, thus, rejected being recruited via social media. Moreover, only 20 (10.3%) out of 195 participants reported an acceptance score >11, indicating high acceptance. These findings indicate that recruitment success via social media might be limited among patients with hepatitis B in Germany and underline the importance of using multiple recruitment channels to facilitate diversity and equitable health care access, particularly for patient groups considered vulnerable [11].

Contrary to what we had hypothesized, SMA was not associated with digital literacy (rejecting H2), data privacy needs (rejecting H5b), and perceived hepatitis B–related stigma (rejecting H6), although reported secrecy around hepatitis B diagnosis was a predictor (confirming H5a). Moreover, trust in medical information sources and demographic variables (age and education) as well as the overall frequency of using social media were not associated with SMA. The results for H2 and H4b are not surprising, as the preceding qualitative interviews did not explicitly indicate a linear connection between digital literacy and social media recruitment acceptance. Our study cannot exclude the possibility that there might be a potential nonlinear association, but another survey study also found that digital literacy did not directly affect the intention to use digital technology [25]. Furthermore, trust is a multifaceted concept [26,27], which is why the subjects of trust were split into medical information sources and other advertisement channels. Hence, it is not unexpected that trust in medical information sources is not associated with SMA.

The rejection of H5b (data privacy) was more surprising, particularly because the qualitative interviews indicated strong connections between data privacy and SMA. In addition, the scholarly debate around data privacy issues has been very salient: data ethicists have repeatedly emphasized the issues related to data privacy and transparency in the context of social media use in the research context [12,28,29]. In addition, the European General Data Protection Regulation emphasizes the transparent use of data and the rights of data subjects [30]. Moreover, various scandals (eg, related to the US presidential election in 2016 and the UK Brexit referendum) diminished users’ trust in social media platforms and increased awareness of data privacy in that context [31,32]. A recent population survey conducted in Germany, the United Kingdom, and the United States confirmed high levels of concern regarding data privacy in all included countries [33]. Given these public discussions about social media activities being problematic for data privacy, it is particularly astonishing that data privacy concerns (as operationalized in our study) were not predicting SMA. The findings align with discussions around the privacy paradox. It was confirmed in numerous studies that social media users display limited data protection behavior despite being concerned about their privacy [34-36]. In line with this, the aforementioned scandals have not resulted in a decline in Facebook users [37,38]. Other studies suggest a poor user awareness of online privacy [39] and fatigue in engaging with privacy-related risks [40]. It seems that the surveyed population with hepatitis B in Germany are also affected by this privacy paradox.

The rejection of H6 (association of stigma) was surprising, too, particularly because of the strong association between hepatitis B and stigma in other studies. An Indian survey study found that most surveyed patients with hepatitis B were subject to severe stigma and moderate to severe discrimination, with gender identification as men, unemployment, and illiteracy being predictors of discrimination [6]. Other survey studies from Australia, Turkey, and Serbia confirmed the presence of self-reported perception of stigma in 35% to 47% of patients with hepatitis B and 60% to 65% of patients with hepatitis C [10,41,42]. An Iranian qualitative study found that patients with hepatitis B conceptualized stigma as both extrinsic (eg, discrimination, public embarrassment, or blame) and intrinsic (eg, perceived rejection, social isolation, and frustration) [8]. Although this empirical evidence illustrates the relative importance of stigma in the context of hepatitis B, this did not predict patients’ acceptance of social media recruitment in our study. Instead, our findings suggest that the perceived secrecy of a hepatitis B diagnosis, which seems to be unrelated to the perception of stigma, is informative on social media recruitment acceptance. This indicates that perceptions of stigma in other stigmatized diseases (eg, sexually transmitted diseases, and psychiatric disorders) might not influence patient acceptance to be recruited via social media for clinical studies. However, empirical studies within these populations need to confirm this.

### Limitations and Further Research

Our survey showed a relatively balanced representation of genders. This aligns with a German serological study from 2011, which indicated no statistically significant difference in the prevalence of acute or chronic hepatitis B infection in men and women [43]. In terms of age distribution, the survey study covered a diverse range of age groups, mirroring the distribution found in the German serological study [43]. On the basis of these observations, the survey sample overall is representative of the population with hepatitis B in Germany regarding gender and age.

However, it is essential to consider potential limitations and sources of bias. The recruitment strategy used, primarily relying on venue-based recruitment within a clinical setting, might introduce selection bias, as it may not fully capture the diverse population that may exist outside such settings. In addition, only 30.4% (285/939) of estimated incoming patients received the questionnaire, which might introduce an additional selection bias. We attempted to mitigate this by explicitly briefing the study nurses to avoid self-selection when distributing the survey. The low distribution rate has been mainly caused by administrative burden, resulting in weeks during which no questionnaires were distributed. Thus, we do not expect this to have a large impact on selection bias.

In addition, the study’s restriction to the German language may have impaired the accessibility of the questionnaire for participants who do not have German as their mother tongue. In addition, the exclusive focus on a German setting may limit the generalizability of the findings to a broader international context, potentially impacting the study’s external validity. Finally, it is important to note that we have shortened the questionnaire in comparison to its original length after discussion with clinical colleagues, who provided the feedback that the questionnaire was too long. As part of this shortening, some validated scales were replaced by self-developed scales, which may have implications for the comprehensiveness and depth of the data collected.

Consequently, the attitudes of patients in other medical conditions toward social media recruitment, and a comparison to the attitudes of patients with hepatitis B assessed in this study, should be subject to further research. Similarly, it will be important to study how the different social media platforms, their underlying logic, use patterns, and other factors might influence patients’ acceptance of social media recruitment over time.

### Conclusions

This study provides the first quantitative data on the acceptance of social media as a recruitment channel for clinical studies. In the context of hepatitis B in Germany, acceptance of being recruited via social media was very limited. More than 1 (28.7%) in 4 participants rejected this recruitment channel. The study sets out to be a reference point for future studies assessing the attitudes and acceptance of social media recruitment for clinical studies. Such empirical inquiries can facilitate the work of researchers designing clinical studies as well as ethics review boards in balancing the risks and benefits of social media recruitment in a context-specific manner. Moreover, this study provides guidance for researchers considering using social media recruitment and ethics review boards judging such undertakings, by cautioning against the potentially low acceptance rates social media–based recruitment might yield for some patient populations. These should be weighed against the risks of social media recruitment for the target populations.

Similarly relevant for practice, the findings indicate that social media recruitment is particularly accepted in patient populations with high interest in participating in clinical studies. This is particularly the case for diseases with insufficient treatment options and historically neglected diseases with high unmet needs [44]. Using social media as a recruitment channel for studies targeting these patient groups might thus encounter higher acceptance levels than in this study. There was no statistically significant role associated with perceived stigma and data privacy needs among patients, suggesting that these concerns are unrelated to social media recruitment acceptance.

## Appendix

Multimedia Appendix 1 Response rate information.

Multimedia Appendix 2 Questionnaire.

Multimedia Appendix 3 Assumptions checks for regression analyses.

Multimedia Appendix 4 Description of each item of the questionnaire.

## Abbreviations

SMA: social media acceptance
