# Supplementary material for: Acceptance of Social Media Recruitment for Clinical Studies Among Patients With Hepatitis B: Mixed Methods Study
Source: J Med Internet Res. 2024 Aug 26;26:e54034. doi: 10.2196/54034 (PMC11384172; doi:10.2196/54034)
Supplement: Multimedia Appendix 1 [file jmir_v26i1e54034_app1.docx]

### Multimedia Appendix 1: Response rate information

|  | **Munich** | **Hannover** | **Leipzig** | **Total** |
| --- | --- | --- | --- | --- |
| Data collection period | 4 June 2022 - 30 April 2023  (11 months) | 23 Aug 2022 –  17 April 2023  (8 months) | 17 Mar 2023 –  31 May 2023 (2,5 months) | 4 June 2022 – 31 May 2023 (12 months) |
| Total no of incoming HepB patients during data collection period | 308 (28 per month) | 576 (72 per month) | 55 (22 per month) | 939 |
| Response rate I (based on incoming patients) | 16,9% | 25,9% | 10,9% | 22,0% |
| Total no of questionnaires distributed | 127 | 152 | 6 | 285 |
| Response rate I (based on no. of questionnaires distributed) | 40,9% | 98,0% | 100,0% | 72,6% |
| Questionnaires received | 52 | 149 | 6 | 207 |
| Questionnaires excluded due to lack of consent | -7 | -5 | 0 | -12 |
| Included questionnaires | 45 | 144 | 6 | 195 |
