# Supplementary material for: Acceptance of Social Media Recruitment for Clinical Studies Among Patients With Hepatitis B: Mixed Methods Study
Source: J Med Internet Res. 2024 Aug 26;26:e54034. doi: 10.2196/54034 (PMC11384172; doi:10.2196/54034)
Supplement: Multimedia Appendix 2 [file jmir_v26i1e54034_app2.pdf]

Teilnahme an klinischen Studien: Neue Wege der Anwerbung

Sehr geehrte Patientin, sehr geehrter Patient,

herzlichen Dank, dass Sie bei dieser Studie mitmachen. Ihre Teilnahme ist sehr wertvoll für uns. Wir wenden uns an Sie, weil Sie an Hepatitis B leiden. In dieser Studie untersuchen wir, wie Hepatitis B Betroffene neue Formen der Anwerbung für klinische Studien einschätzen. Bitte lesen Sie vor dem Ausfüllen dieses Fragebogens die beigelegte Studieninformation sorgfältig durch. Bei Fragen wenden Sie sich an das Klinikpersonal oder die Studienkoordinatorin Theresa Willem: theresa.willem@tum.de, Tel. +49 89 4140 4041

☐ **Ich habe die Information gelesen und bin mit der Verarbeitung meiner Daten einverstanden**
Bitte beachten Sie: Ohne dieses Kreuz dürfen wir Ihren Fragebogen nicht für die Studie verwenden und sind datenschutzrechtlich verpflichtet, diesen zu vernichten.

Danke für Ihr Einverständnis. Das Ausfüllen des Fragebogens dauert ungefähr 10 Minuten. Bitte lesen Sie alles sorgfältig, bevor Sie antworten. Bitte füllen Sie den Fragebogen möglichst vollständig aus, das ist sehr wichtig für die Aussagekraft der Studie.
Soziale Medien sind digitale Plattformen zum Austausch von Informationen und Lebensinhalten. Sie werden auch manchmal zur Anwerbung von Teilnehmenden für wissenschaftliche Studien genutzt. Zunächst möchten wir erfahren, welche Sozialen Medien Sie wie häufig nutzen. Wie häufig nutzen Sie die folgenden Sozialen Medien?

		Nie	Einmal im Monat oder seltener	Zwei- bis viermal im Monat	Mehrmals in der Woche	Mehrmals täglich
1.01	WhatsApp	☐	☐	☐	☐	☐
1.02	Telegram	☐	☐	☐	☐	☐
1.03	Youtube	☐	☐	☐	☐	☐
1.04	Facebook	☐	☐	☐	☐	☐
1.05	Instagram	☐	☐	☐	☐	☐
1.06	Pinterest	☐	☐	☐	☐	☐
1.07	Twitter	☐	☐	☐	☐	☐
1.08	Xing	☐	☐	☐	☐	☐
1.09	LinkedIn	☐	☐	☐	☐	☐
1.10	Snapchat	☐	☐	☐	☐	☐
1.11	Reddit	☐	☐	☐	☐	☐
1.12	TikTok	☐	☐	☐	☐	☐
1. 13	Tumblr	☐	☐	☐	☐	☐

Als nächstes interessiert uns, wie gut Sie sich mit den sozialen Medien auskennen. Wie stark würden Sie den folgenden Aussagen zustimmen?

		Stimme gar nicht zu	Stimme eher nicht zu	Stimme teilweise zu	Stimme eher zu	Stimme voll und ganz zu
2.01	Ich weiß, wie ich ein Konto in den sozialen Medien erstelle.	☐	☐	☐	☐	☐
2.02	Ich weiß, wie ich mein Konto in den sozialen Medien lösche.	☐	☐	☐	☐	☐
2.03	Ich weiß, wie ich mein Konto in den sozialen Medien deaktiviere.	☐	☐	☐	☐	☐

		Stimme gar nicht zu	Stimme eher nicht zu	Stimme teilweise zu	Stimme eher zu	Stimme voll und ganz zu
2.04	Ich weiß, wie ich Inhalte wie Fotos in meinem Konto in den sozialen Medien teile.	☐	☐	☐	☐	☐
2.05	Ich weiß, wie ich unerwünschte Inhalte aus meinem Konto in den sozialen Medien entferne.	☐	☐	☐	☐	☐
2.06	Ich kenne die Copyright-Gesetze, denen die sozialen Medien unterliegen.	☐	☐	☐	☐	☐
2.07	Ich weiß, wie ich Konflikten in den sozialen Medien angemessen begegne.	☐	☐	☐	☐	☐
2.08	Ich kenne die Richtlinien für soziale Medien in meinem beruflichen Umfeld.	☐	☐	☐	☐	☐
2.09	Ich weiß, wie ich den Wahrheitsgehalt der in den sozialen Medien geteilten Informationen überprüfe.	☐	☐	☐	☐	☐
2.10	Ich weiß, wie ich verschiedene Informationsquellen zur Überprüfung von Informationen aus den sozialen Medien nutzen kann.	☐	☐	☐	☐	☐
2.11	Ich kann einschätzen, ob eine Information in den sozialen Medien wahr oder falsch ist.	☐	☐	☐	☐	☐
2.12	Plattformen wie Facebook steuern, was ich in den sozialen Medien sehe.	☐	☐	☐	☐	☐
2.13	Informationen, die ich in sozialen Medien poste, sind dauerhaft.	☐	☐	☐	☐	☐
2.14	Die Werbung, die ich in den sozialen Medien sehe, ist speziell auf meine Vorlieben ausgerichtet.	☐	☐	☐	☐	☐

Bisher ging es darum, wie Sie die sozialen Medien *generell* nutzen. Im folgenden Abschnitt soll es nun um die Nutzung sozialer Medien in Bezug auf Ihre Hepatitis B Erkrankung gehen. Wie häufig nutzen Sie die folgenden Sozialen Medien in Bezug auf Ihre Hepatitis B Erkrankung? *Falls Sie keine sozialen Medien nutzen, kreuzen Sie bitte an, welche Anwendungsbereiche Sie sich grundsätzlich vorstellen könnten.*

		Nie	Selten	Gelegent-lich	Häufig	Sehr häufig
3.01	Zum Austausch mit anderen Betroffenen.	☐	☐	☐	☐	☐
3.02	Um verlässliche medizinische Informationen zu finden.	☐	☐	☐	☐	☐
3.03	Um auf dem neuesten Stand der Forschung zu bleiben.	☐	☐	☐	☐	☐
3.04	Um von wissenschaftlichen Studien zu neuen Hepatitis B Behandlungen zu erfahren.	☐	☐	☐	☐	☐
3.05	Um Kontakte zu vertrauenswürdigen Wissenschaftlern und Studienleitern zu knüpfen.	☐	☐	☐	☐	☐
3.06	Um behandelnde Ärzt:innen zu finden.	☐	☐	☐	☐	☐

Klinische Studien sind wissenschaftliche Studien zur Erforschung neuer medizinischer Behandlungen. In diesem Teil des Fragebogens geht es um die Anwerbung von Teilnehmer:innen für klinische Hepatitis B Studien. Zunächst geht es nun darum, wie interessiert Sie grundsätzlich an der Teilnahme an einer klinischen Studie sind. Wie stark würden Sie den folgenden beiden Aussagen zustimmen?

		Stimme gar nicht zu	Stimme eher nicht zu	Stimme teilweise zu	Stimme eher zu	Stimme voll und ganz zu
4.01	Ich bin allgemein bereit an klinischen Studien teilzunehmen.	☐	☐	☐	☐	☐
4.02	Die Teilnahme an klinischen Studien zu Hepatitis B ist mir wichtig.	☐	☐	☐	☐	☐

Nun interessiert uns für wie vertrauenswürdig sie verschiedene Quellen halten, um erstmals von einer klinischen Hepatitis B Studie zu erfahren. Für wie vertrauenswürdig halten Sie die Information über eine klinische Hepatitis B Studie, wenn Sie sie von dieser Quelle erhalten?

		Gar nicht	Eher nicht	Teil-weise	Eher schon	Sehr
5.01	Behandelnder Arzt / Ärztin	☐	☐	☐	☐	☐
5.02	Andere medizinische Fachperson (Pflegende, Assistent:innen, administratives Klinikpersonal etc.)	☐	☐	☐	☐	☐
5.03	Andere Patienten	☐	☐	☐	☐	☐
5.04	Plakatwerbung in der Öffentlichkeit	☐	☐	☐	☐	☐
5.05	Zeitungsinserate	☐	☐	☐	☐	☐
5.06	Werbespots im Fernsehen	☐	☐	☐	☐	☐
5.07	Online Plattform (z.B. eine spezialisierte Plattform, die klinische Studien präsentiert)	☐	☐	☐	☐	☐
5.08	Studienspezifische Webseite	☐	☐	☐	☐	☐
5.09	Soziale Medien: Werbebanner	☐	☐	☐	☐	☐
5.10	Soziale Medien: Persönliche Nachricht von einem Ihnen unbekannten Absender	☐	☐	☐	☐	☐
5.11	Soziale Medien: Persönliche Nachricht von der Studienleitung	☐	☐	☐	☐	☐

Im nächsten Abschnitt geht es um Ihre Einstellung gegenüber sozialen Medien zur Anwerbung für klinische Hepatitis B Studien. Wie stark würden Sie den folgenden Aussagen zustimmen?

		Stimme gar nicht zu	Stimme eher nicht zu	Stimme teilweise zu	Stimme eher zu	Stimme voll und ganz zu
6.01	Soziale Medien eignen sich gut, um Patient:innen auf Studien zu neuen Hepatitis B Behandlungen aufmerksam zu machen.	☐	☐	☐	☐	☐
6.02	Soziale Medien steigern die Erfolgsaussichten von klinischen Hepatitis B Studien.	☐	☐	☐	☐	☐
6.03	Ich würde mich über soziale Medien für eine klinische Hepatitis B Studie anwerben lassen.	☐	☐	☐	☐	☐
6.04	Ich würde soziale Medien nutzen, um mich über klinische Hepatitis B Studien zu informieren.	☐	☐	☐	☐	☐
6.05	Es ist schwierig für mich, geeignete Kanäle zu finden um mich über klinische Studien zu Hepatitis B zu informieren.	☐	☐	☐	☐	☐

Sie haben es bald geschafft! In diesem Teil der Umfrage geht es um Ihre Bedenken bezüglich Ihrer Privatsphäre im Zusammenhang mit Ihrer Hepatitis B Erkrankung. Bitte beantworten Sie für jede Frage, wie stark sie auf Sie zutrifft.

		Stimme gar nicht zu	Stimme eher nicht zu	Stimme teilweise zu	Stimme eher zu	Stimme voll und ganz zu
7.01	Meine Familie und Freunde wissen von meiner Hepatitis B Erkrankung.	☐	☐	☐	☐	☐
7.02	Meine Hepatitis B Erkrankung ist ein Geheimnis.	☐	☐	☐	☐	☐
7.03	Ich achte darauf, dass ich in den sozialen Medien nichts über meine Hepatitis B Erkrankung bekannt gebe, weil ich befürchte, dass die Plattform diese Informationen sammelt und speichert.	☐	☐	☐	☐	☐
7.04	Ich möchte, dass meine medizinischen Daten im Zusammenhang mit meiner Erkrankung besonders gut geschützt werden.	☐	☐	☐	☐	☐

Zudem interessiert uns Ihre soziale Befindlichkeit in Bezug auf Ihre Hepatitis B Erkrankung. Manche der folgenden Fragen gehen davon aus, dass andere von Ihrer Hepatitis B Erkrankung wissen. Das trifft auf Sie vielleicht nicht zu. Stellen Sie sich in dem Fall bitte vor, Sie würden sich in dieser Situation befinden. Wie stark würden Sie den folgenden Aussagen zustimmen?

		Stimme gar nicht zu	Stimme eher nicht zu	Stimme teilweise zu	Stimme eher zu	Stimme voll und ganz zu
8.01	Die Reaktion anderer Menschen auf meine Hepatitis B Infektion hat mich verletzt.	☐	☐	☐	☐	☐
8.02	Manche Menschen vermeiden es, mich zu berühren, sobald sie von meiner Hepatitis B Infektion erfahren haben.	☐	☐	☐	☐	☐
8.03	Manche Menschen wollen mich nicht mehr in der Nähe ihrer Kinder haben, sobald sie von meiner Hepatitis B Infektion erfahren.	☐	☐	☐	☐	☐
8.04	Andere Menschen sind körperlich vor mir zurückgewichen, als sie erfuhren, dass ich Hepatitis B habe.	☐	☐	☐	☐	☐
8.05	Ich habe aufgrund ihrer Reaktionen auf meine Hepatitis B Erkrankung aufgehört, mich mit manchen Leuten zu treffen.	☐	☐	☐	☐	☐
8.06	Die Leute scheinen Angst vor mir zu haben, sobald sie von meiner Hepatitis B Infektion erfahren.	☐	☐	☐	☐	☐

Zum Abschluss bitten wir Sie um einige persönliche Angaben. Diese sind sehr wichtig für die Auswertung der Studie. Die Daten werden datenschutzkonform verarbeitet und gespeichert.

Wie alt sind Sie? __________

Mit welchem Geschlecht identifizieren Sie sich?

- ☐ Männlich
- ☐ Weiblich
- ☐ Divers
- ☐ Keine Angabe

Was ist ihr höchster Bildungsabschluss?

- ☐ Noch Schüler:in
- ☐ Schule beendet ohne Abschluss
- ☐ Hauptschulabschluss
- ☐ Realschulabschluss
- ☐ Abschluss einer Fachoberschule, etc. (Fachhochschulreife)
- ☐ Abgeschlossene Berufsausbildung
- ☐ Abitur (Hochschulreife)
- ☐ Hochschulabschluss (abgeschlossenes Studium)
- ☐ Anderen Ausbildungsabschluss, und zwar: ______________________
- ☐ Keine Angabe

Was ist Ihre Muttersprache?

Mehrfachantworten möglich

- ☐ Deutsch
- ☐ Andere
- ☐ Keine Angabe

Herzlichen Dank für Ihre Teilnahme!

Bitte geben Sie nun den Fragebogen beim Klinikpersonal ab. **Bitte nehmen Sie die Patienteninformation (erster Bogen) mit nach Hause und bewahren ihn sicher auf.** Auf dem Bogen finden Sie Ihre persönliche Kennungsnummer. Bitte geben Sie diese an, wenn Sie Ihre Daten einzusehen, sperren oder löschen lassen möchten. Da wir keine Kontaktdaten erheben, ist eine Verarbeitung Ihrer
