# Supplementary material for: Acceptance of Social Media Recruitment for Clinical Studies Among Patients With Hepatitis B: Mixed Methods Study
Source: J Med Internet Res. 2024 Aug 26;26:e54034. doi: 10.2196/54034 (PMC11384172; doi:10.2196/54034)
Supplement: Multimedia Appendix 3 [file jmir_v26i1e54034_app3.docx]

### Multimedia Appendix 3: Assumptions checks for regression analyses

Cook’s distance indicated that the data contained no outliers (highest value = .682), suggesting no individual cases were unduly influencing the model. Collinearity statistics (Tolerance, VIF) indicated that multicollinearity was not a concern. Durbin-Watson test indicated that the data met the assumption of independent errors (Durbin-Watson = 2.073). Normal P-P plot of standardized residuals indicated that the data contained normally distributed errors (great majority of points on the line). Scatterplots showed that the relationship between the independent variables and the dependent variable is linear (relationships characterized by a straight line; note, three variables had to be dichotomized to meet this assumption); scatterplots of standardized predicted values showed that the data met the assumption of homoscedasticity (looking like a random array of dots). The data also met the assumption of non-zero variances.
