# Supplementary material for: Acceptance of Social Media Recruitment for Clinical Studies Among Patients With Hepatitis B: Mixed Methods Study
Source: J Med Internet Res. 2024 Aug 26;26:e54034. doi: 10.2196/54034 (PMC11384172; doi:10.2196/54034)
Supplement: Multimedia Appendix 4 [file jmir_v26i1e54034_app4.docx]

### Multimedia Appendix 4: Description of each questionnaire item

| **Item** | | **N** | | **Min** | **Max** | **Mean** | **Median** | **Std deviation** |
| --- | --- | --- | --- | --- | --- | --- | --- | --- |
|  |  | **Valid** | **Missing** |  |  |  |  |  |
| **Frequency of social media usage** | | | | | | | | |
| P1.1 | Whatsapp | 195 | 0 | 0 | 4 | 3,37 | 4 | 1,286 |
| P1.2 | Telegram | 195 | 0 | 0 | 4 | 0,49 | 0 | 1,073 |
| P1.3 | Youtube | 195 | 0 | 0 | 4 | 2,24 | 3 | 1,347 |
| P1.4 | Facebook | 195 | 0 | 0 | 4 | 1,48 | 1 | 1,660 |
| P1.5 | Instagram | 195 | 0 | 0 | 4 | 1,32 | 0 | 1,744 |
| P1.6 | Pinterest | 195 | 0 | 0 | 4 | 0,47 | 0 | 0,949 |
| P1.7 | Twitter | 195 | 0 | 0 | 4 | 0,25 | 0 | 0,802 |
| P1.8 | Xing | 195 | 0 | 0 | 4 | 0,22 | 0 | 0,672 |
| P1.9 | LinkedIn | 195 | 0 | 0 | 4 | 0,31 | 0 | 0,830 |
| P1.10 | Snapchat | 195 | 0 | 0 | 4 | 0,25 | 0 | 0,832 |
| P1.11 | Reddit | 195 | 0 | 0 | 4 | 0,13 | 0 | 0,577 |
| P1.12 | TikTok | 195 | 0 | 0 | 4 | 0,64 | 0 | 1,291 |
| P1.13 | Tumblr | 195 | 0 | 0 | 4 | 0,06 | 0 | 0,421 |
| **Social media literacy** | | | | | | | | |
| P2.01 | I know how to create a social media account. | 194 | 1 | 0 | 4 | 3,00 | 4 | 1,443 |
| P2.02 | I know how to delete my social media account. | 193 | 2 | 0 | 4 | 2,82 | 3 | 1,448 |
| P2.03 | I know how to deactivate my social media account. | 193 | 2 | 0 | 4 | 2,78 | 3 | 1,449 |
| P2.04 | I know how to share content such as fotos in my social media account. | 190 | 5 | 0 | 4 | 3,16 | 4 | 1,234 |
| P2.05 | I know how to remove content from my social media account. | 188 | 7 | 0 | 4 | 2,83 | 3 | 1,349 |
| P2.06 | I know the copyright laws relevant to social media. | 185 | 10 | 0 | 4 | 2,12 | 2 | 1,362 |
| P2.07 | I know how to meet conflicts on social media | 188 | 7 | 0 | 4 | 2,46 | 3 | 1,337 |
| P2.08 | I know the social media guidelines in my professional activities. | 189 | 6 | 0 | 4 | 2,47 | 3 | 1,413 |
| P2.09 | I know how to verify the truthfulness of information shared on social media. | 188 | 7 | 0 | 4 | 2,51 | 3 | 1,346 |
| P2.10 | I know how to use different information sources to verify information from social media. | 187 | 8 | 0 | 4 | 2,73 | 3 | 1,281 |
| P2.11 | I can assess whether information on social media is true or false | 188 | 7 | 0 | 4 | 2,61 | 3 | 1,186 |
| P2.12 | Platforms like Facebook control what I see on social media. | 186 | 9 | 0 | 4 | 2,31 | 2,5 | 1,563 |
| P2.13 | Information I post on social media is permanent. | 186 | 9 | 0 | 4 | 2,40 | 3 | 1,408 |
| P2.14 | The ads I see on social media are specifically targeted to my preferences. | 186 | 9 | 0 | 4 | 2,55 | 3 | 1,403 |
| **Social media usage for hepatitis B** | | | | | | | | |
| P3.01 | To exchange with other patients | 189 | 6 | 0 | 4 | 0,38 | 0 | 0,923 |
| P3.02 | To find reliable medical information | 188 | 7 | 0 | 4 | 1,26 | 1 | 1,214 |
| P3.03 | To keep up with the latest research results | 186 | 9 | 0 | 4 | 1,09 | 1 | 1,161 |
| P3.04 | To learn about new clinical studies for new treatment options for Hepatitis B. | 188 | 7 | 0 | 4 | 1,09 | 1 | 1,209 |
| P3.05 | To get in touch with trustworthy researchers and study coordinators. | 187 | 8 | 0 | 4 | 0,51 | 0 | 0,986 |
| P3.06 | To find treating physicians. | 184 | 11 | 0 | 4 | 0,98 | 0 | 1,310 |
| **Willingness to participate in clinical trials** | | | | | | | | |
| P4.01 | I am generally willing to participate in clinical trials | 188 | 7 | 0 | 4 | 2,65 | 3 | 1,313 |
| P4.02 | Participating in clinical trials for Hepatitis B is important to me. | 190 | 5 | 0 | 4 | 2,88 | 3 | 1,259 |
| **Trusted information sources** | | | | | | | | |
| P5.01 | Treating physician | 188 | 7 | 0 | 4 | 3,54 | 4 | 0,861 |
| P5.02 | Other medical professionals (nurses, assistants, administrative hospital staff, etc.) | 183 | 12 | 0 | 4 | 2,78 | 3 | 1,213 |
| P5.03 | Other patients | 181 | 14 | 0 | 4 | 1,56 | 2 | 1,122 |
| P5.04 | Public poster ads | 183 | 12 | 0 | 4 | 1,26 | 1 | 1,117 |
| P5.05 | Newspaper ads | 185 | 10 | 0 | 4 | 1,27 | 1 | 1,095 |
| P5.06 | TV ads | 185 | 10 | 0 | 4 | 1,16 | 1 | 1,100 |
| P5.07 | Online platforms (specialized clinical trial platforms) | 184 | 11 | 0 | 4 | 1,89 | 2 | 1,234 |
| P5.08 | Study-specific website | 183 | 12 | 0 | 4 | 2,08 | 2 | 1,309 |
| P5.09 | Social media ads | 184 | 11 | 0 | 4 | 0,95 | 1 | 0,977 |
| P5.10 | Social media: Personal message from an unknown source | 184 | 11 | 0 | 4 | 0,44 | 0 | 0,820 |
| P5.11 | Social media: Personal message from the study leader | 182 | 13 | 0 | 4 | 1,67 | 2 | 1,258 |
| **Acceptance of social media recruitment** | | | | | | | | |
| P6.01 | Social media are well suited to make patients aware of studies on new hepatitis B treatments. | 184 | 11 | 0 | 4 | 1,99 | 2 | 1,226 |
| P6.02 | Social media increase the likelihood of success in hepatitis B clinical trials. | 180 | 15 | 0 | 4 | 1,81 | 2 | 1,123 |
| P6.03 | I would be recruited via social media for a hepatitis B clinical trial. | 181 | 14 | 0 | 4 | 1,13 | 1 | 1,135 |
| P6.04 | I would use social media to learn about hepatitis B clinical trials. | 182 | 13 | 0 | 4 | 1,58 | 2 | 1,227 |
| P6.05 | It is difficult for me to find appropriate channels to learn about hepatitis B clinical trials. | 184 | 11 | 0 | 4 | 1,97 | 2 | 1,276 |
| **Secrecy** | | | | | | | | |
| P7.01 | My family and friends know about my Hepatitis B infection. | 188 | 7 | 0 | 4 | 3,05 | 4 | 1,155 |
| P7.02 | My hepatitis B infection is a secret. | 186 | 9 | 0 | 4 | 1,30 | 1 | 1,288 |
| **Data privacy** | | | | | | | | |
| P7.03 | I am careful not to disclose anything about my hepatitis B infection on social media for fear that the platform will collect and store this information. | 186 | 9 | 0 | 4 | 2,73 | 3 | 1,516 |
| P7.04 | I want my medical data in connection with my illness to be particularly well protected. | 189 | 6 | 0 | 4 | 3,52 | 4 | 0,908 |
| **Perceived stigma** | | | | | | | | |
| P8.01 | Other people's reactions to my hepatitis B infection hurt me. | 186 | 9 | 0 | 4 | 1,39 | 1 | 1,303 |
| P8.02 | Some people avoid touching me as soon as they find out about my my hepatitis B infection. | 186 | 9 | 0 | 4 | 1,05 | 1 | 1,223 |
| P8.03 | Some people don't want me around their children once they learn about my hepatitis B infection. | 185 | 10 | 0 | 4 | 0,82 | 0 | 1,091 |
| P8.04 | Other people physically backed away from me when they learned that I had hepatitis B. | 185 | 10 | 0 | 4 | 0,85 | 0 | 1,125 |
| P8.05 | I stopped seeing some people because of their reactions to my hepatitis B disease. | 185 | 10 | 0 | 4 | 0,65 | 0 | 1,152 |
| P8.06 | People seem to be afraid of me as soon as they find out about my hepatitis B infection. | 183 | 12 | 0 | 4 | 0,92 | 0 | 1,202 |

Notes: Items were measured through a 5-point Likert Scale, ranging from 0 (completely disagree) to 4 (completely agree). The original questionnaire was in German.
